# Supplementary material for: [1,2,4]Triazolo[3,4-b]benzothiazole Scaffold as Versatile Nicotinamide Mimic Allowing Nanomolar Inhibition of Different PARP Enzymes
Source: J Med Chem. 2023 Jan 4;66(2):1301–20. doi: 10.1021/acs.jmedchem.2c01460 (PMC9884089; doi:10.1021/acs.jmedchem.2c01460)
Supplement: Supplementary file 1 — jm2c01460_si_001.pdf [file jm2c01460_si_001.pdf]

Supplementary Information

[1,2,4]Triazolo[3,4-*b*]benzothiazole scaffold as versatile nicotinamide mimic allowing nanomolar inhibition of different PARP enzymes

Sudarshan Murthy[1,#], Maria Giulia Nizi[2,#], Mirko M. Maksimainen[1,#], Serena Massari[2], Juho Alaviuhkola[1], Barbara E. Lippok[3], Chiara Vagaggini[4], Sven T. Sowa[1], Albert Galera-Prat[1], Yashwanth Ashok[1], Harikanth Venkannagari[1], Renata Prunskaite-Hyyryläinen[1], Elena Dreassi[4], Bernhard Lüscher[3], Patricia Korn[3], Oriana Tabarrini[2,*] & Lari Lehtiö[1,*]

[1]Faculty of Biochemistry and Molecular Medicine & Biocenter Oulu, University of Oulu, 90220 Oulu, Finland

[2]Department of Pharmaceutical Sciences, University of Perugia, 06123 Perugia, Italy

[3]Institute of Biochemistry and Molecular Biology, RWTH Aachen University, 52074 Aachen, Germany

[4]Department of Biotechnology, Chemistry and Pharmacy, University of Siena, I-53100, Siena, Italy.

*Address correspondence to OT: E-mail: Oriana.tabarrini@unipg.it or LL: E-mail: lari.lehtio@oulu.fi

#These authors contributed equally

CONTENT

Table S1. Crystallography data processing and refinement statistics.

Figure S1. WST-1 cell toxicity analysis of compounds.

Figure S2. Binding modes of OUL40 analogs to TNKS2 and PARP15.

Figure S3. Examples of IC_{50} measurements.

Figure S4. Dose dependence of the PARP10 rescue assay.

Example of HPLC analysis of the target compounds

Example of ^{1}HNMR and ^{13}CNMR spectra of the target compounds

Table S1. Data collection and refinement statistics.

	TNKS2-**1** (PDB 7R3Z)	PARP14-**1** (PDB 7R3L)	PARP15-**1** (PDB 7R3O)	PARP15-**8** (PDB 7R4A)
**Data collection**				
Beamline	DLS, I04	ESRF, ID30A-1	DLS, I04	MAX IV, BioMax
Wavelength (Å)	0.97949	0.96600	0.97950	0.979957
Space group	$C222_1$	C2	$P2_12_12_1$	$P2_12_12_1$
Cell dimensions				
a, *b*, *c* (Å)	91.96, 97.49, 117.90	81.88, 83.75, 80.03	45.24, 68.54, 158.57	45.46, 68.74, 159.23
α, β, γ (°)	90, 90, 90	90, 115.7, 90	90, 90, 90	90, 90, 90
Resolution (Å)	50 – 2.25	53 – 1.999	50 – 2.20	50 – 1.90
Outer shell (Å)	2.31 – 2.25	2.033 – 1.999	2.26 – 2.20	1.95 – 1.90
No. unique reflections	25467 (1844)	32725 (1587)	25834 (1893)	40198 (2893)
R_{merge}	0.123 (1.51)	0.088 (0.534)	0.185 (0.942)	0.145 (1.25)
Mean $I/\sigma I$	10.7 (1.96)	10.3 (2.30)	7.80 (1.94)	8.1 (1.80)
CC ½ (%)	99.6 (74.5)	99.7 (84.2)	99.4 (79.1)	99.6 (46.2)
Completeness (%)	99.9 (99.9)	99.2 (99.2)	99.9 (100)	99.9 (99.9)
Redundancy	6.5 (6.8)	3.1 (3.1)	6.6 (6.7)	6.4 (6.6)
**Refinement**				
R_{work} / R_{free}	0.197 / 0.245	0.202 / 0.237	0.205 / 0.242	0.233 / 0.272
No. atoms				
Protein	3352	2683	3193	3183
Ligand/ion	54	41	17	18
Water	78	145	88	150
B-factors				
Protein	46.1	24.7	34.2	33.5
Ligand/ion	49.04	33.2	42.6	35.0
Water	39.3	25.7	26.0	36.6
R.m.s. deviations				
Bond lengths (Å)	0.0065	0.0073	0.0080	0.0088
Bond angles (°)	1.448	1.418	1.515	1.528
Ramachandran plot (%)				
Favored	97.8	98.7	97.2	97.9
Allowed	2.2	1.3	2.8	2.1
Outliers	0	0	0	0

Values in parentheses are for highest-resolution shell.

Table S1. Data collection and refinement statistics. Contd.

	TNKS2-**3** (PDB 7R5X)	PARP15-**6** (PDB 7R5D)	PARP15-**7** (PDB 7Z1W)	PARP15-**16** (PDB 7Z1Y)
**Data collection**				
Beamline	DLS I03	ESRF, ID30A-1	ESRF, ID30A-1	ESRF, ID30A-1
Wavelength (Å)	0.92023	0.96546	0.96546	0.96546
Space group	$C222_1$	$P2_12_12_1$	$P2_12_12_1$	$P2_12_12_1$
Cell dimensions				
a, *b*, *c* (Å)	91.94, 96.99, 119.44	45.37, 68.80, 160.94	45.33, 68.47, 159.91	45.23, 68.33, 158.65
α, β, γ (°)	90, 90, 90	90, 90, 90	90, 90, 90	90, 90, 90
Resolution (Å)	48.49 - 2.0	50 – 2.15	50 – 1.90	50 – 1.75
Outer shell (Å)	2.072 - 2.0	2.21 – 2.15	1.95 – 1.90	1.80 – 1.75
No. unique reflections	36379 (3591)	27222 (1843)	39946 (2903)	50369 (3652)
R_{merge}	0.0953 (0.722)	0.057 (0.292)	0.112 (0.855)	0.096 (0.968)
Mean $I/\sigma I$	15.2 (3.46)	14.5 (3.73)	9.0 (1.81)	12.6 (2.25)
CC ½ (%)	99.8 (87.1)	99.7 (93.5)	99.6 (70.6)	99.8 (74.7)
Completeness (%)	99.93 (100.0)	96.4 (91.1)	99.5 (99.9)	99.6 (99.2)
Redundancy	7.9 (8.4)	3.7 (2.8)	4.9 (4.7)	6.3 (5.9)
**Refinement**				
R_{work} / R_{free}	0.189 / 0.223	0.203 / 0.249	0.180 / 0.212	0.226 / 0263
No. atoms				
Protein	3408	3182	3208	3202
Ligand/ion	54	19	17	17
Water	169	135	202	121
B-factors				
Protein	37.9	31.9	29.4	27.9
Ligand/ion	42.5	51.7	44.1	47.4
Water	37.8	31.2	33.1	29.8
R.m.s. deviations				
Bond lengths (Å)	0.013	0.0072	0.0076	0.0088
Bond angles (°)	1.7	1.50	1.46	1.58
Ramachandran plot				
Favored	98.5	97.2	98.5	97.2
Allowed	1.5	2.5	1.5	2.8
Outliers	0	0.3	0	0

Values in parentheses are for highest-resolution shell.

Table S1. Data collection and refinement statistics. Contd.

	PARP2-**16** (PDB 7R59)	PARP15-**11** (PDB 7Z1V)	PARP15-**14** (PDB 7Z41)	PARP15-**13** (PDB 7Z2O)	PARP15-**27** (PDB 7Z2Q)
**Data collection**					
Beamline	DLS, I03	ESRF, ID30A-1	DLS, I04	DLS, I03	ESRF, ID30A-1
Wavelength	0.97625	0.96546	0.97950	0.9763	0.96546
Space group	$P2_12_12_1$	$P2_12_12_1$	$P2_12_12_1$	$P2_12_12_1$	$P2_12_12_1$
Cell dimensions					
a, *b*, *c* (Å)	58.38, 67.82, 86.69	45.14, 68.38, 159.16	45.28, 68.61, 158.84	45.31, 68.80, 160.21	45.24, 68.67, 159.88
α, β, γ (°)	90, 90, 90	90, 90, 90	90, 90, 90	90, 90, 90	90, 90, 90
Resolution (Å)	50-2.00	50 – 1.50	50 – 2.10	50 – 1.50	50 – 2.00
Outer shell (Å)	2.05-2.00	1.54 – 1.50	2.10 – 2.15	1.54 – 1.50	2.05 – 2.00
No. unique reflections	23900 (1743)	79300 (5774)	29760 (2184)	81152 (5903)	34370 (2523)
R_{merge}	0.235 (1.405)	0.082 (0.798)	0.181 (0.714)	0.050 (1.279)	0.148 (0.771)
Mean $I/\sigma I$	9.47 (1.90)	9.6 (1.79)	6.7 (1.7)	26.1 (2.09)	6.3 (1.84)
CC ½ (%)	99.6 (72.6)	99.7 (69.1)	99.4 (78.5)	100 (81.0)	98.9 (70.2)
Completeness (%)	100 (100)	99.3 (99.6)	99.9 (99.9)	100 (100)	99.4 (99.8)
Redundancy	13.4 (13.9)	4.2 (4.2)	6.6 (6.3)	13.1 (13.1)	4.0 (4.2)
**Refinement**					
R_{work} / R_{free}	0.187 / 0.238	0.143 / 0.189	0.192 / 0.232	0.148 / 0.187	0.193 / 0.216
No. atoms					
Protein	2764	3253	3203	3262	3202
Ligand/ion	19	22	20	18	21
Water	199	318	202	290	196
B-factors					
Protein	27.3	21.4	33.1	28.6	28.1
Ligand/ion	23.9	25.4	38.6	32.8	29.0
Water	28.9	31.2	34.6	36.1	30.9
R.m.s. deviations					
Bond lengths (Å)	0.0086	0.0097	0.0070	0.0087	0.0062
Bond angles (°)	1.494	1.497	1.481	1.472	1.419
Ramachandran plot					
Favored	97.1	98.5	97.7	98.5	97.7
Allowed	2.6	1.5	2.3	1.5	2.0
Outliers	0.3	0	0	0	0.3

Values in parentheses are for highest-resolution shell.

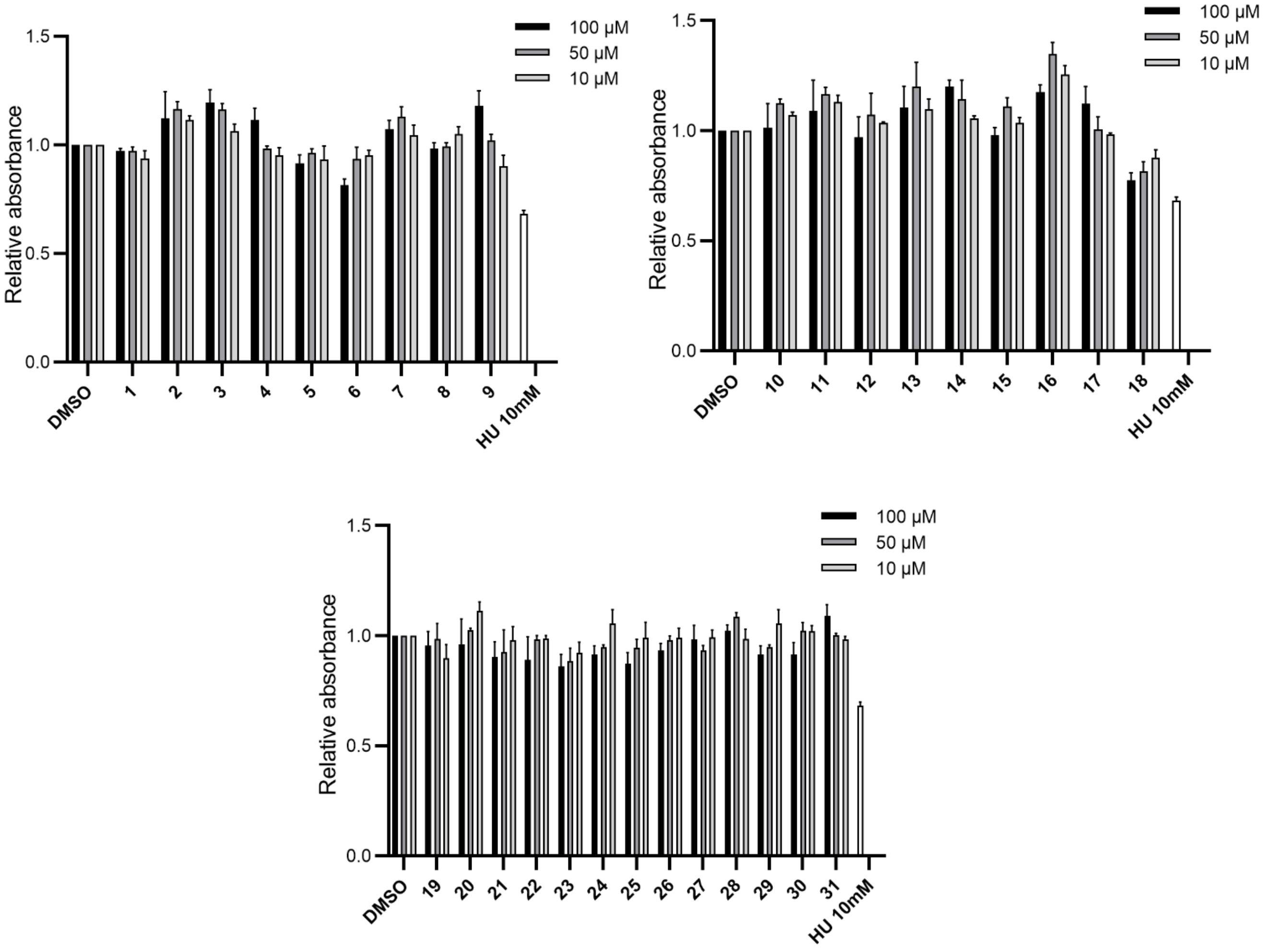

Figure S1. Graph represents cell viability used to determine compound **1-31** toxicity in HEK293T cells. Hydroxyurea (HU, 10 mM) was used as toxicity reference. Data were normalized to DMSO. The bars represent the means ± SEM of three experiment repeats.

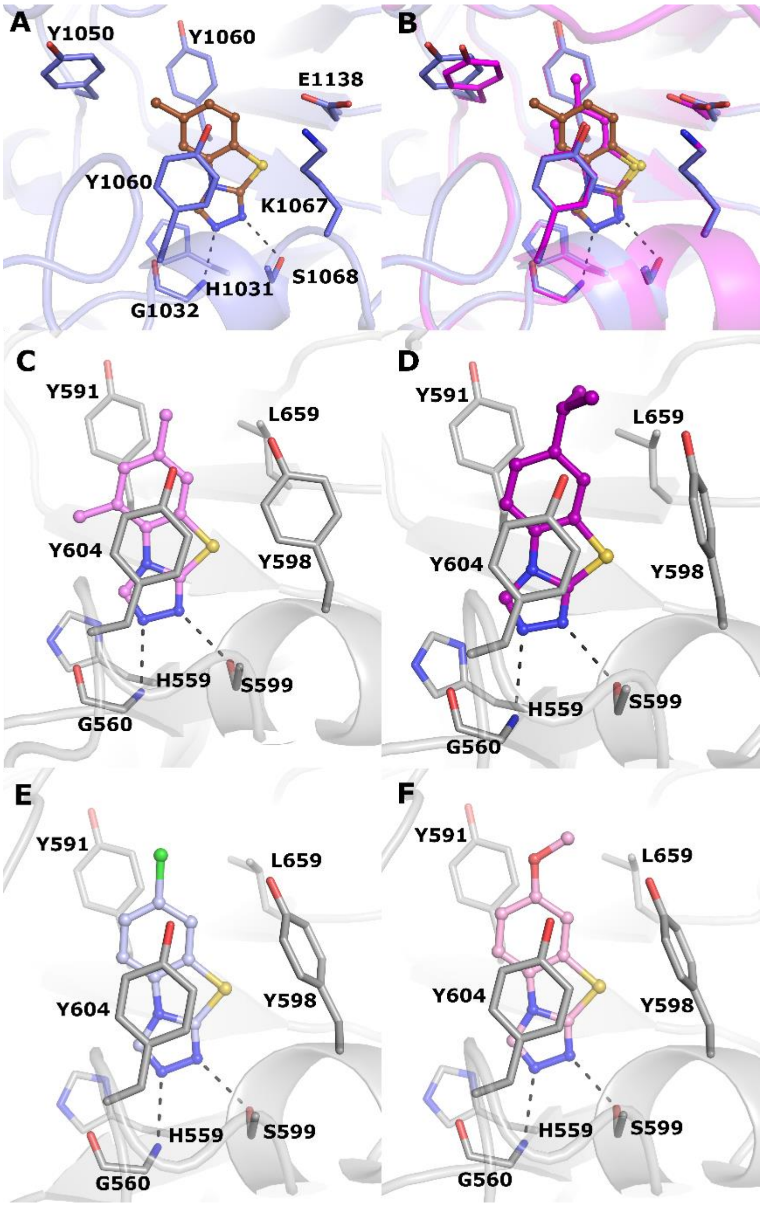

Figure S2. Binding modes of OUL40 analogs to TNKS2 and PARP15. **(A)** TNKS2 crystal structure in complex with **3** (PDB ID 7R5X). The ligand is colored in brown **(B)** Superimposition of the TNKS2 complex structures of **3** and **1**(PDB IDs 7R5X and 7R3Z). The complex structure of **1** is colored in magenta. **(C)** PARP15 crystal structure in complex with **8** (PDB ID 7R4A). The ligand is colored in violet (**D**) PARP15 crystal structure in complex with **6** (PDB ID 7R5D). The ligand is colored in purple (**E**) PARP15 crystal structure in complex with **7** (PDB ID 7Z1W). The ligand is colored in light blue (**F**) PARP15 crystal structure in complex with **11** (PDB ID 7Z1V). The ligand is colored in pink. Hydrogen bonds are indicated in black dash lines.

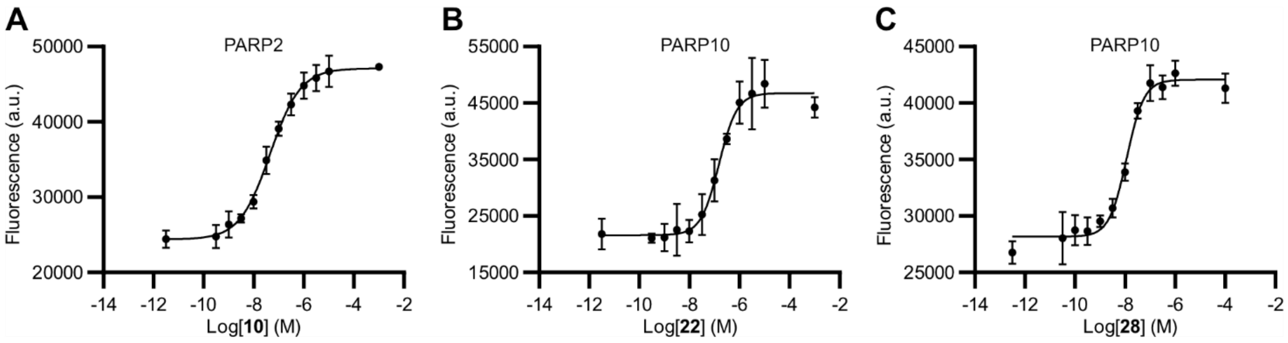

Figure S3. IC_{50} curve examples of the best compounds **16**, **21** and **27**. **(A)** IC_{50} curve of **16** measured with PARP2. **(B)** IC_{50} curve of **21** measured with PARP10. **(C)** IC_{50} curve of **27** measured with PARP10. Data shown are mean from n=4 measurements ± standard deviation. Controls were placed two logarithmic units above the highest or below the lowest compound concentration measured.

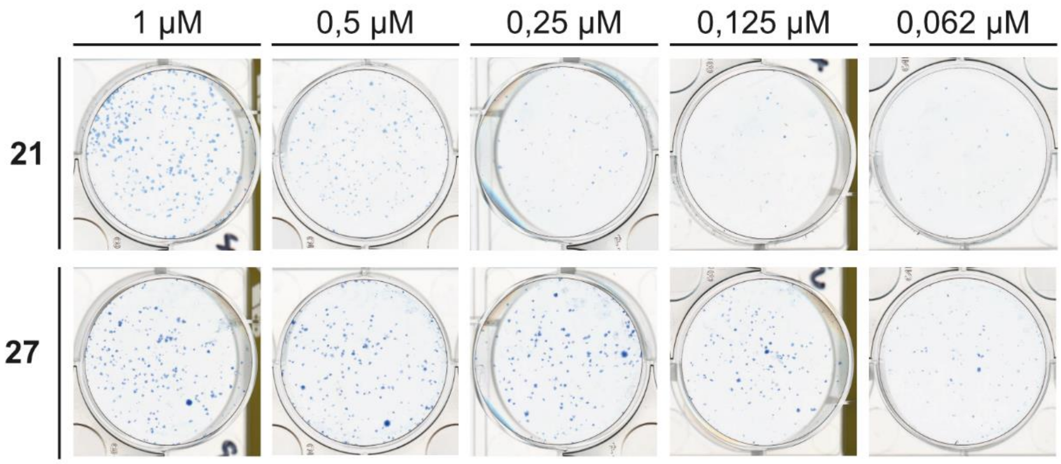

Figure S4. Representative images of the titration experiment related to Figure 5B.

Examples of HPLC analysis of target compounds

6

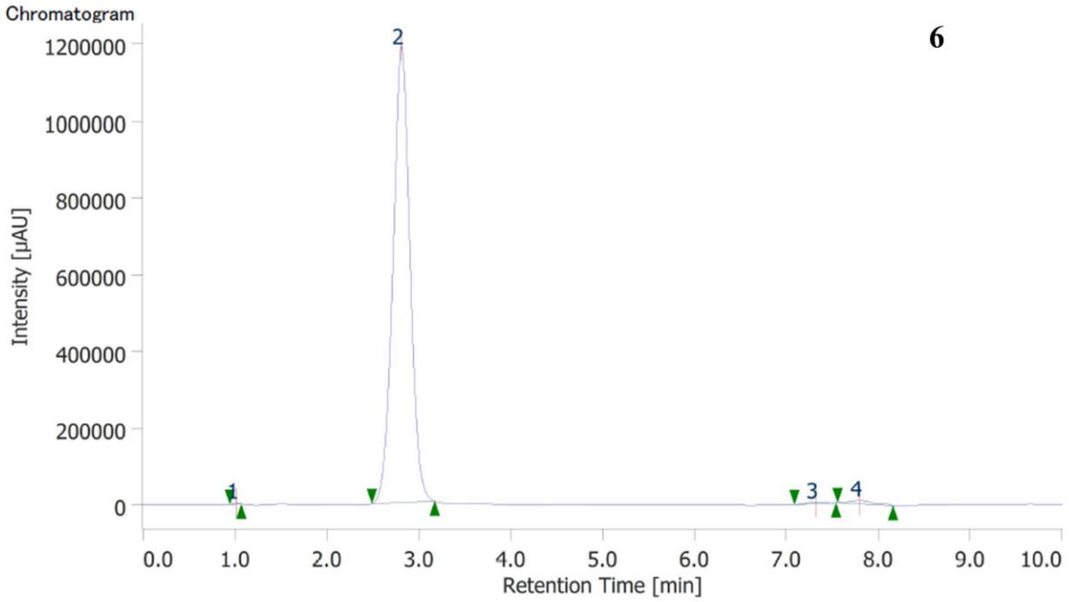

Peak Information

#	Peak Name	CH	tR [min]	Area [µV·sec]	Height [µV]	Area%	Height%	Quantity	Resolution
1	Peak-001	9	1.017	13822	3229	0.089	0.268	N/A	7.946
2	Peak-002	9	2.810	15238258	1188136	98.491	98.723	N/A	11.766
3	Peak-003	9	7.317	55013	3680	0.356	0.306	N/A	1.077
4	Peak-004	9	7.797	164654	8455	1.064	0.703	N/A	N/A

15

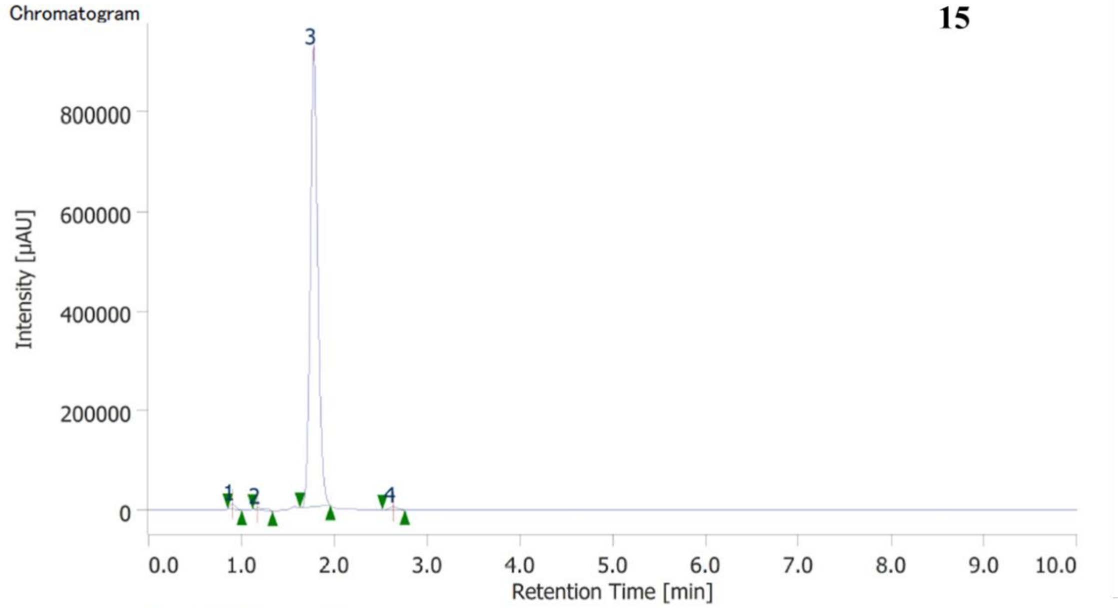

Peak Information

#	Peak Name	CH	tR [min]	Area [µV·sec]	Height [µV]	Area%	Height%	Quantity	Resolution
1	Unknown	9	0.903	41683	9834	0.792	1.043	N/A	1.399
2	Unknown	9	1.180	30953	3429	0.588	0.364	N/A	2.902
3	Unknown	9	1.783	5150668	923460	97.882	97.934	N/A	5.508
4	Unknown	9	2.637	38840	6216	0.738	0.659	N/A	N/A

21

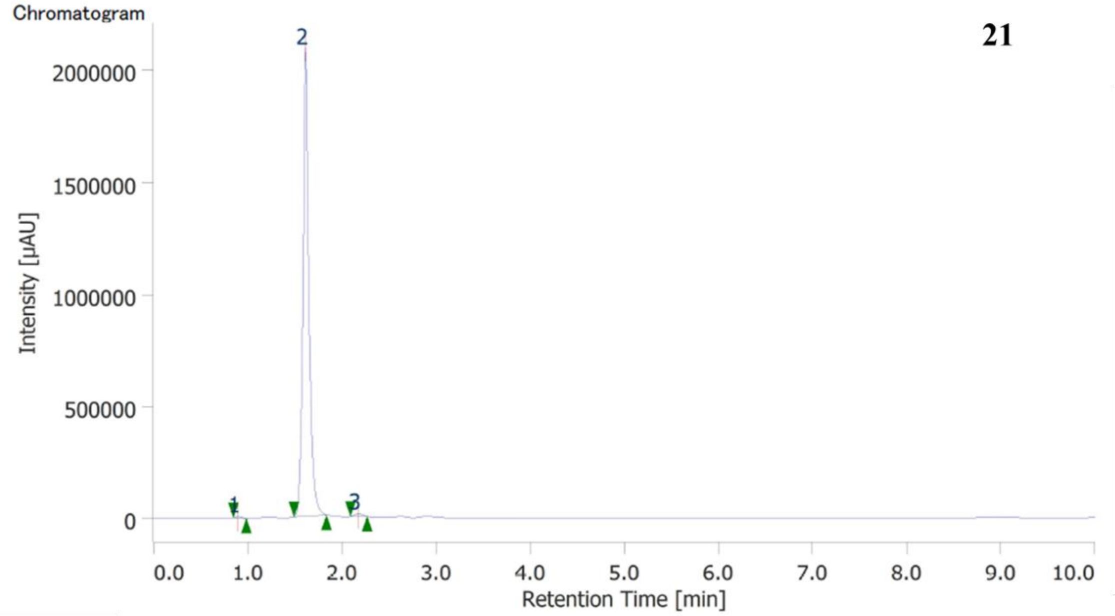

Peak Information

#	Peak Name	CH	tR [min]	Area [µV·sec]	Height [µV]	Area%	Height%	Quantity	Resolution
1	Unknown	9	0.893	23152	5750	0.251	0.272	N/A	6.849
2	Unknown	9	1.613	9125956	2096449	98.979	99.166	N/A	4.155
3	Unknown	9	2.173	70968	11885	0.770	0.562	N/A	N/A

24

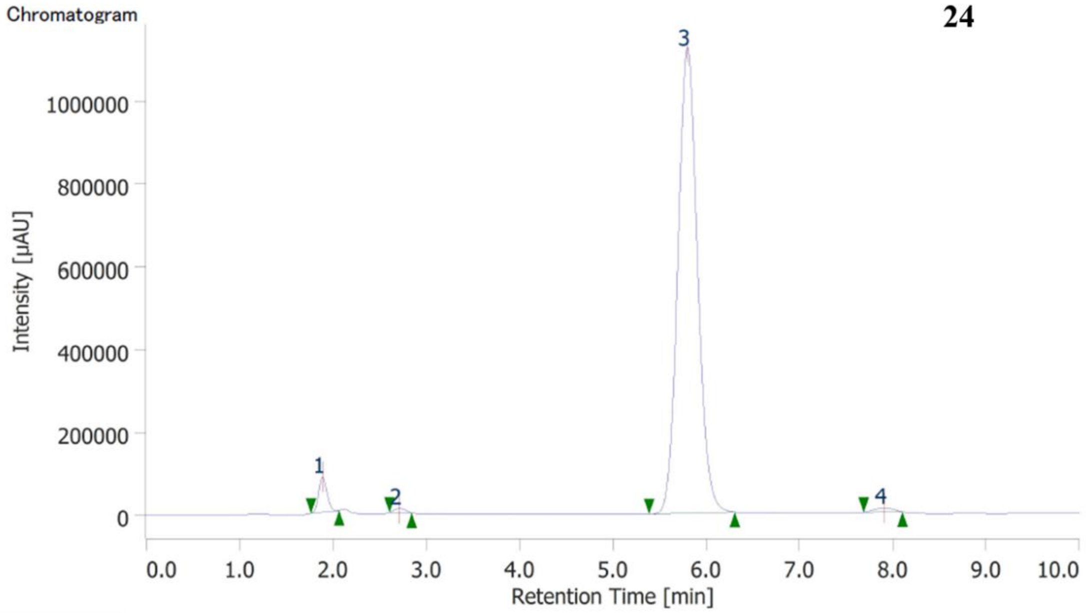

Peak Information

#	Peak Name	CH	tR [min]	Area [µV·sec]	Height [µV]	Area%	Height%	Quantity	Resolution
1	Unknown	9	1.890	526042	85238	3.057	6.925	N/A	4.011
2	Unknown	9	2.710	83441	9870	0.485	0.802	N/A	9.883
3	Unknown	9	5.800	16459689	1125846	95.644	91.471	N/A	5.357
4	Unknown	9	7.907	140164	9864	0.814	0.801	N/A	N/A

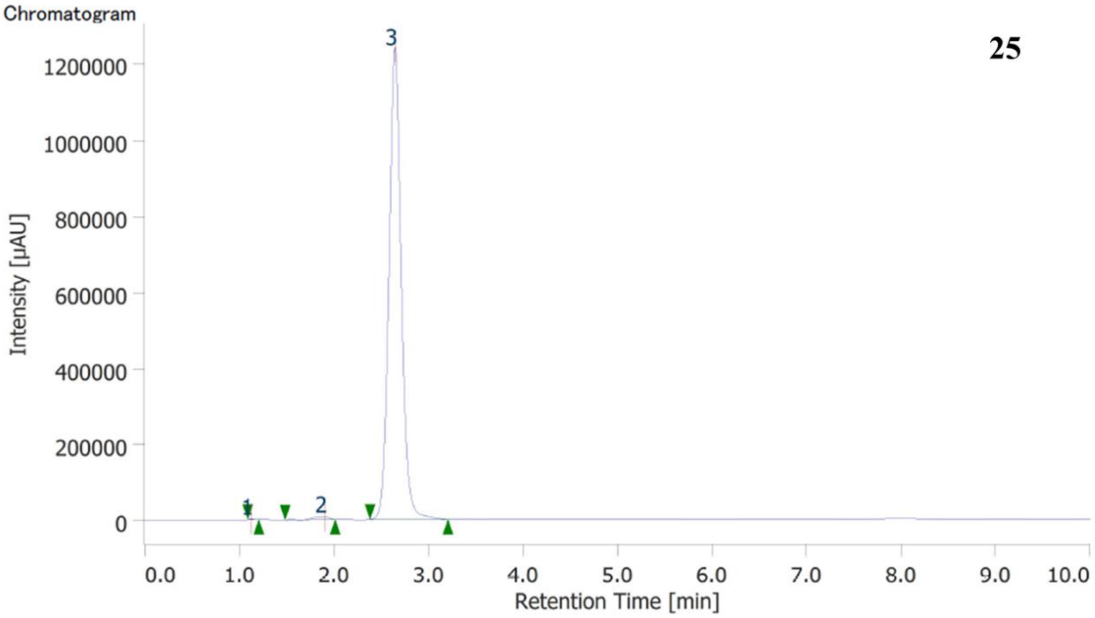

25

Peak Information

#	Peak Name	CH	tR [min]	Area [µV·sec]	Height [µV]	Area%	Height%	Quantity	Resolution
1	Unknown	9	1.123	6608	1983	0.059	0.159	N/A	3.811
2	Unknown	9	1.903	93277	7554	0.830	0.604	N/A	2.689
3	Unknown	9	2.647	11136319	1240213	99.111	99.237	N/A	N/A

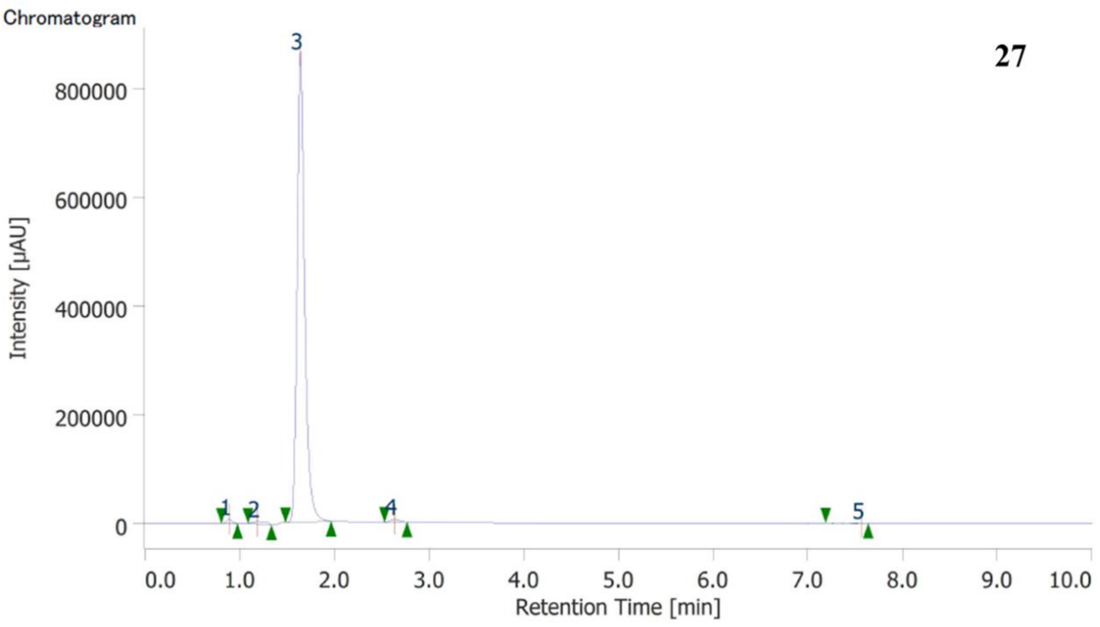

Peak Information

#	Peak Name	CH	tR [min]	Area [µV·sec]	Height [µV]	Area%	Height%	Quantity	Resolution
1	Unknown	9	0.890	31288	7812	0.632	0.885	N/A	1.431
2	Unknown	9	1.190	46221	4465	0.933	0.506	N/A	1.994
3	Unknown	9	1.640	4843949	865625	97.788	98.047	N/A	6.366
4	Unknown	9	2.637	31988	4939	0.646	0.559	N/A	12.250
5	Unknown	9	7.567	100	25	0.002	0.003	N/A	N/A

31

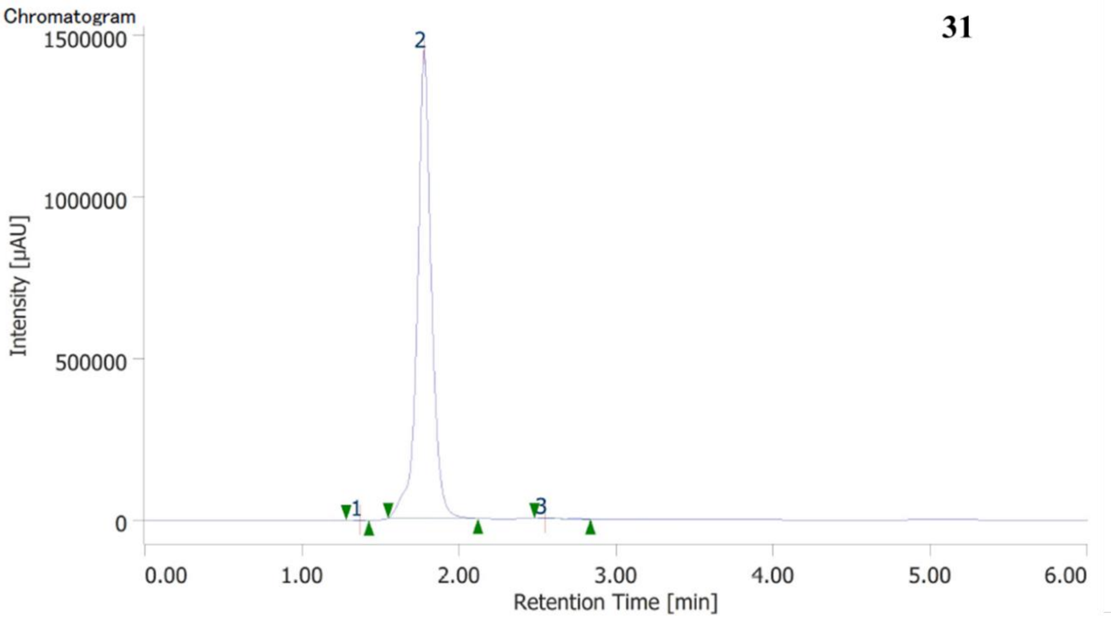

Peak Information

#	Peak Name	CH	tR [min]	Area [µV·sec]	Height [µV]	Area%	Height%	Quantity	Resolution
1	Unknown	9	1.370	6517	1460	0.072	0.100	N/A	3.008
2	Unknown	9	1.777	9056710	1450053	99.764	99.758	N/A	5.186
3	Unknown	9	2.547	14870	2063	0.164	0.142	N/A	N/A

Example of ^{1}HNMR and ^{13}CNMR spectra of target compounds

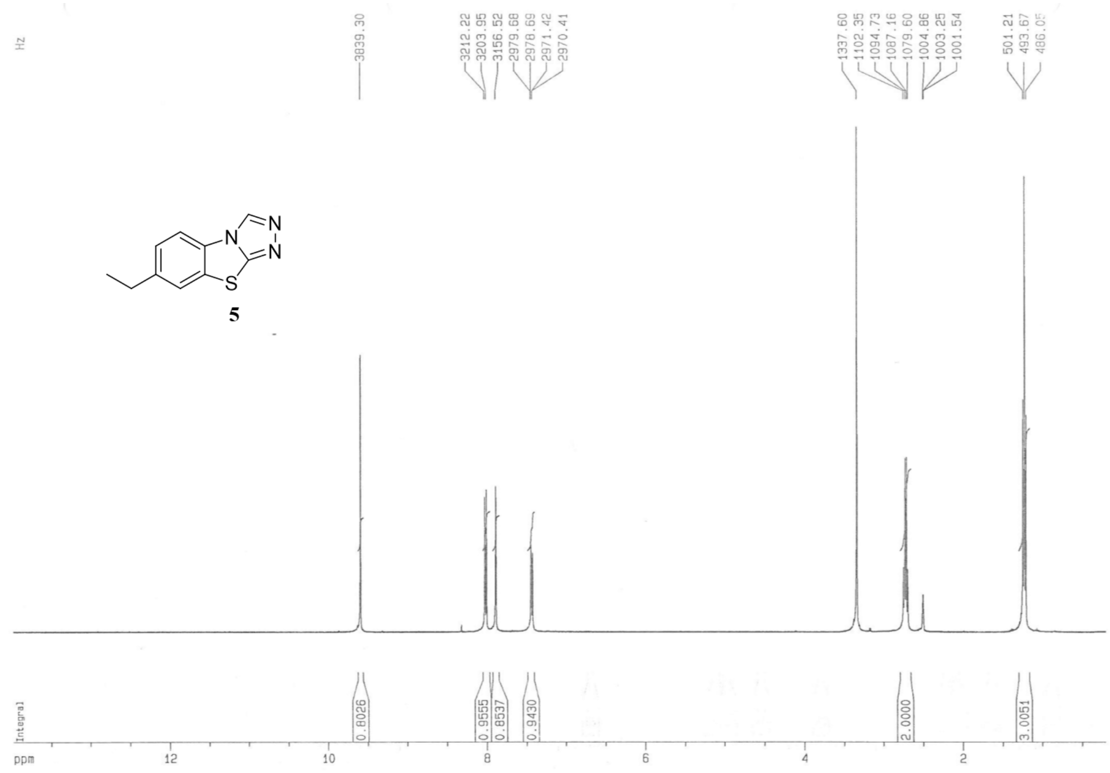

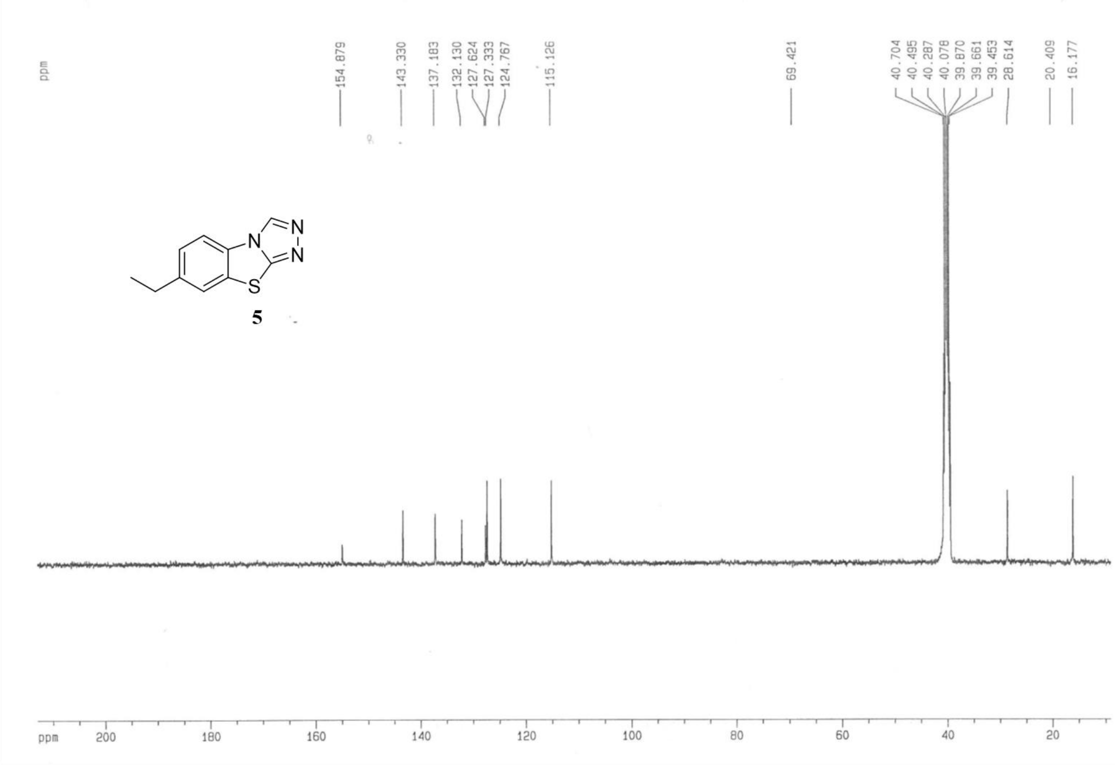
ppm
154.879
143.330
137.183
132.130
127.624
127.333
124.767
115.126
69.421
40.704
40.495
40.287
40.078
39.870
39.661
39.453
28.614
20.409
16.177
5
ppm 200 180 160 140 120 100 80 60 40 20

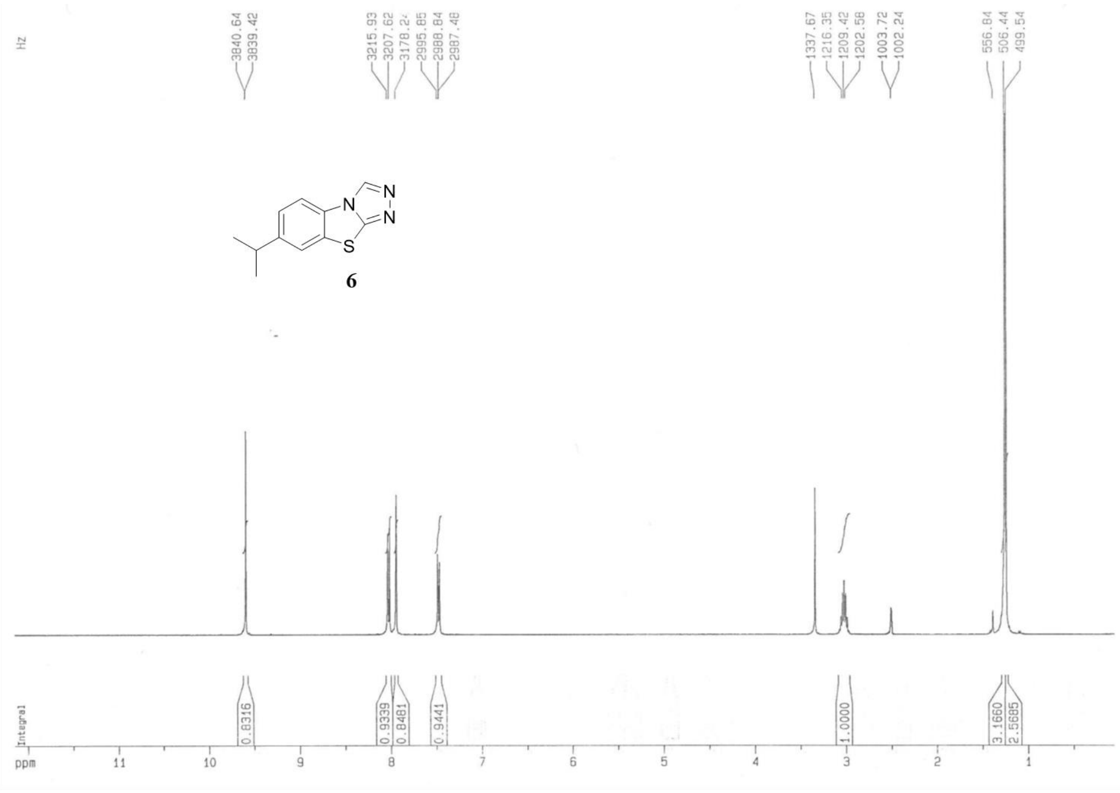
Hz
3840.64
3839.42
3215.93
3207.62
2995.85
2988.84
2987.48
1337.67
1216.35
1209.42
1202.58
1003.72
1002.24
556.84
506.44
499.54
6
Integral
0.8316
0.9339
0.8481
0.9441
1.0000
3.1660
2.5685
ppm 11 10 9 8 7 6 5 4 3 2 1

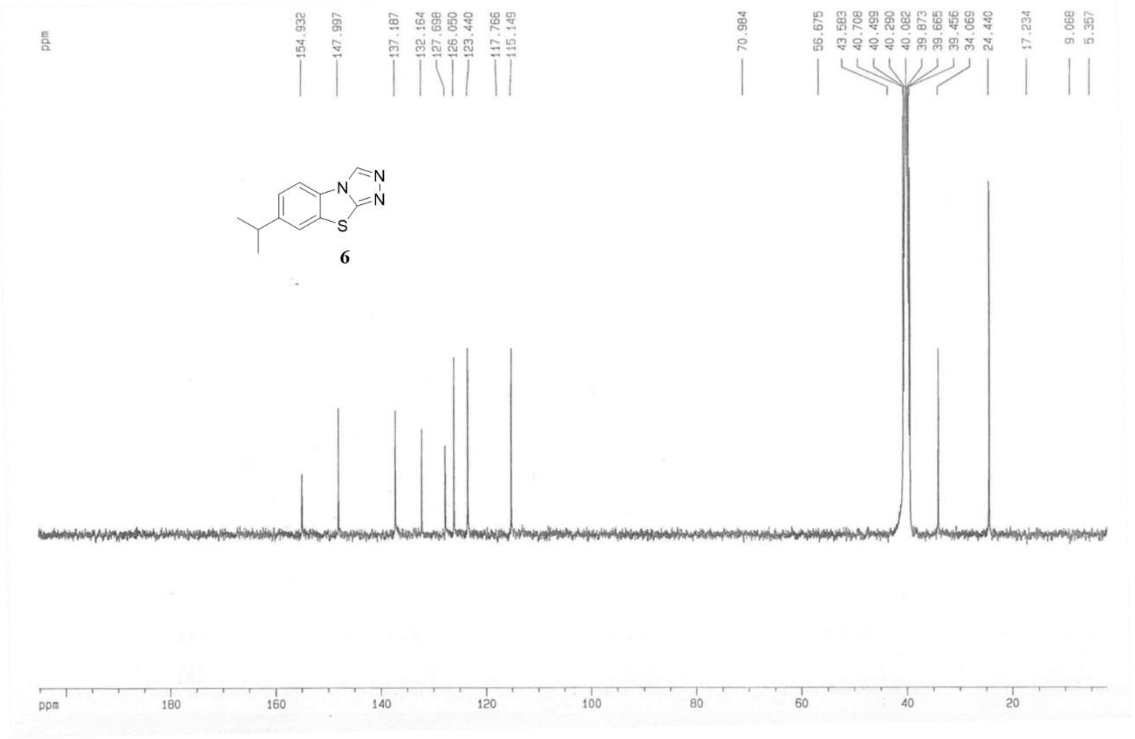
ppm
154.932
147.997
137.187
132.164
127.698
126.050
123.440
117.766
115.149
70.984
56.675
43.583
40.708
40.499
40.290
40.082
39.873
39.665
39.456
34.069
24.440
17.234
9.068
5.357
N
N
N
S
6
ppm 180 160 140 120 100 80 60 40 20

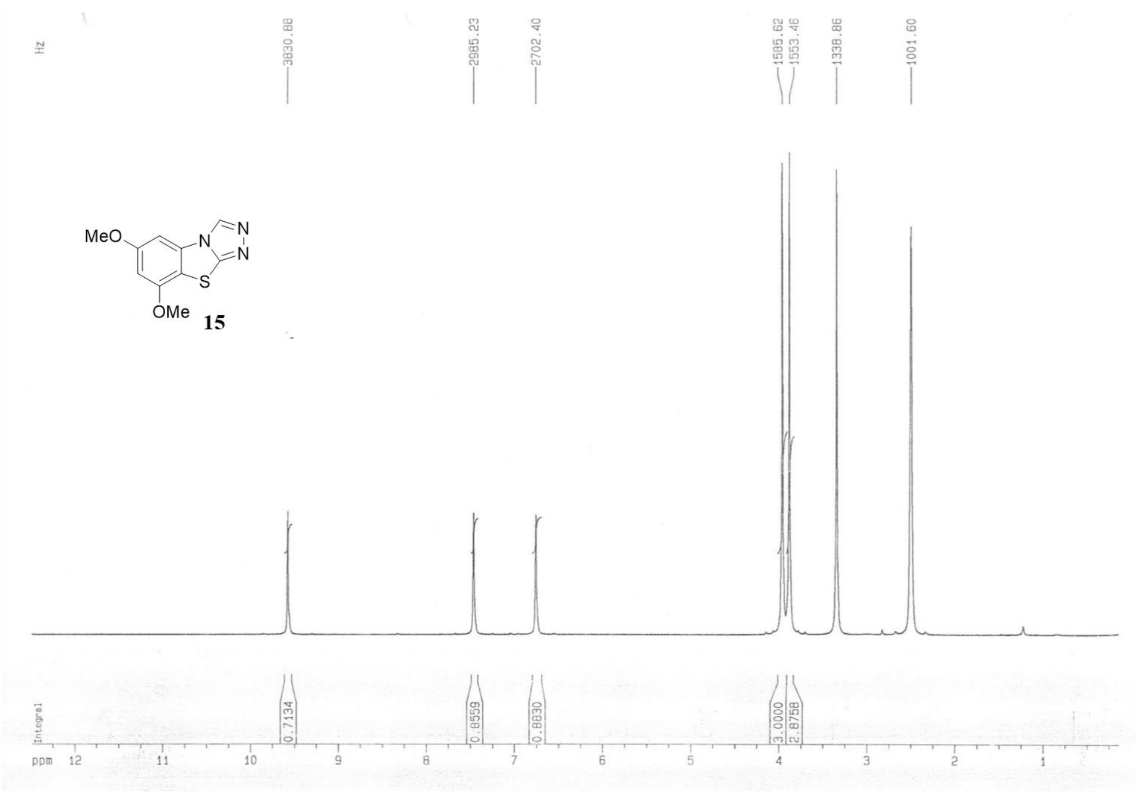
Hz
3830.88
2985.23
2702.40
1585.62
1553.46
1338.86
1001.60
MeO
N
N
N
S
OMe
15
Integral
0.7134
0.8559
0.8830
3.0000
2.8758
ppm 12 11 10 9 8 7 6 5 4 3 2 1

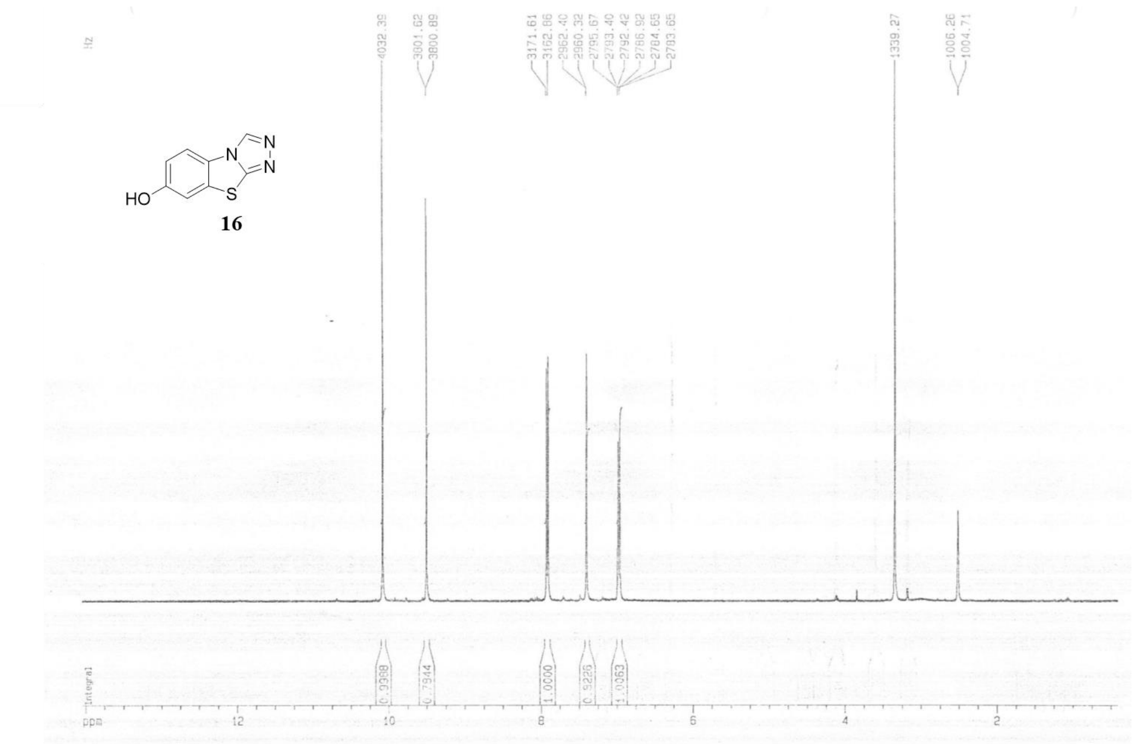
Hz
4032.39
3801.62
3800.89
3171.61
3162.86
2962.40
2960.32
2795.67
2793.40
2792.42
2786.92
2784.65
2783.65
1339.27
1006.26
1004.71
HO
S
N
16
Integral
0.9398
0.7944
1.0000
0.9226
1.0063
ppm
12
10
8
6
4
2

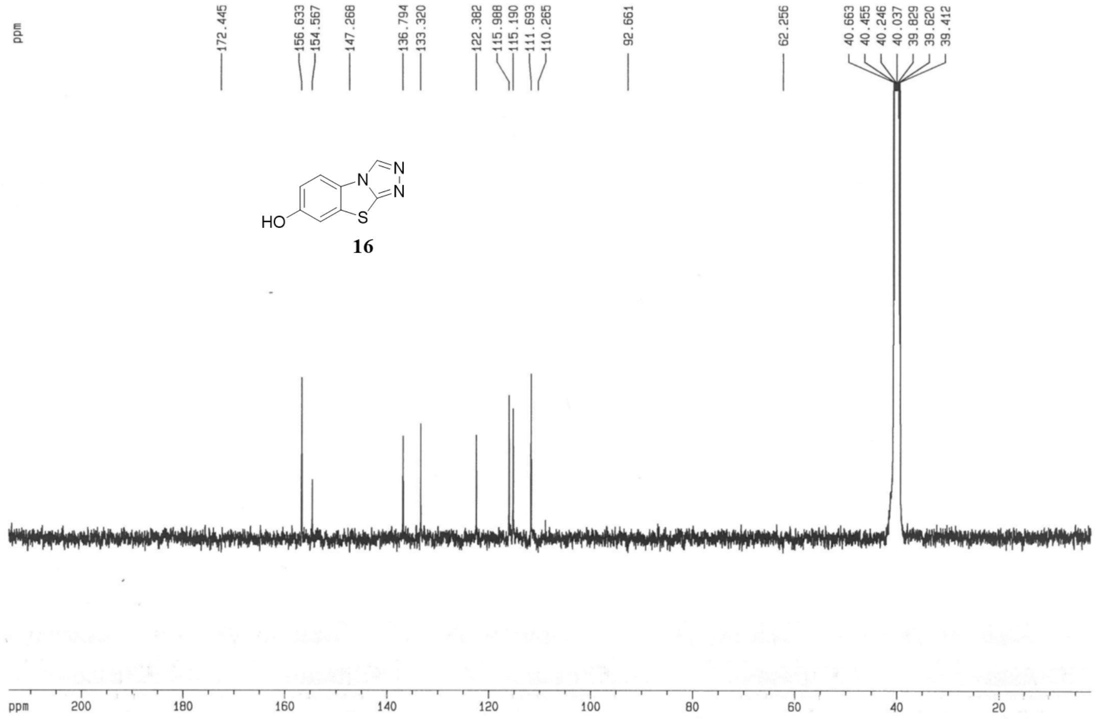
ppm
172.445
156.633
154.567
147.268
136.794
133.320
122.382
115.988
115.190
111.693
110.265
92.661
62.256
40.663
40.455
40.246
40.037
39.829
39.620
39.412
HO
S
N
16
ppm
200
180
160
140
120
100
80
60
40
20

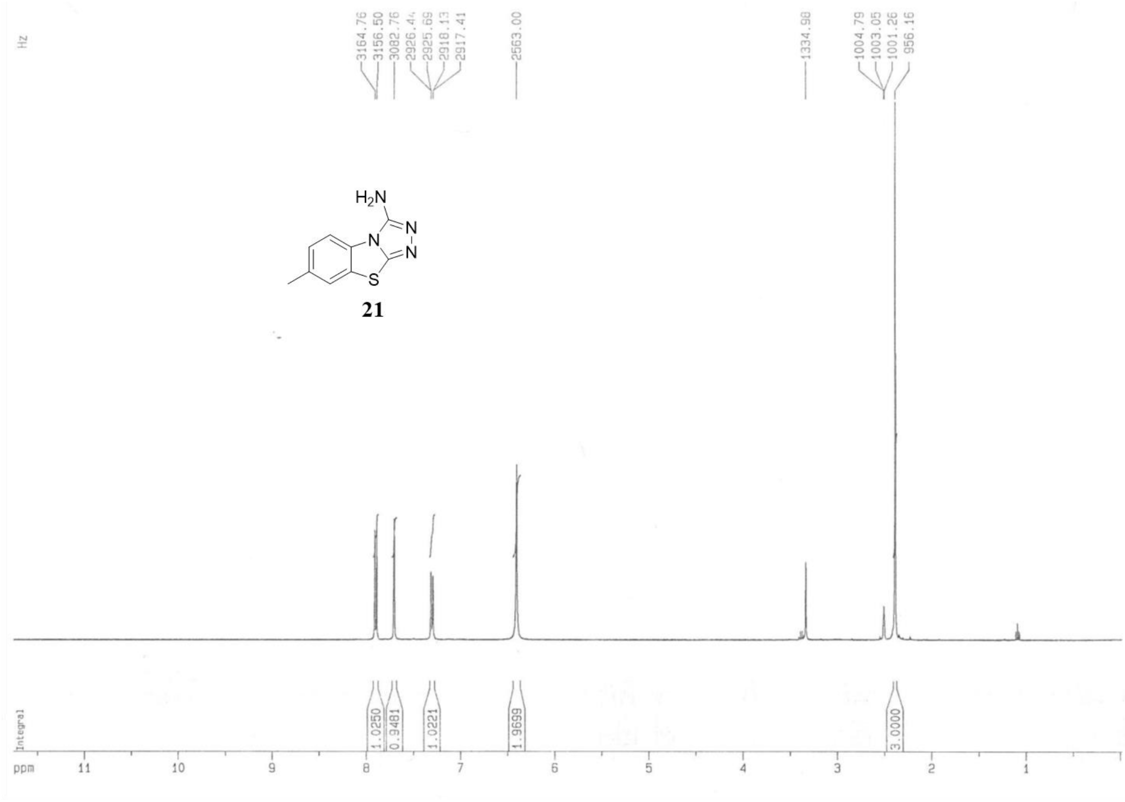
Hz
3164.76
3156.50
3082.76
2926.44
2925.69
2918.13
2917.41
2563.00
1334.98
1004.79
1003.05
1001.26
956.16
H_2N
N
N
N
S
21
Integral
1.0250
0.9481
1.0221
1.9699
3.0000
ppm
11
10
9
8
7
6
5
4
3
2
1

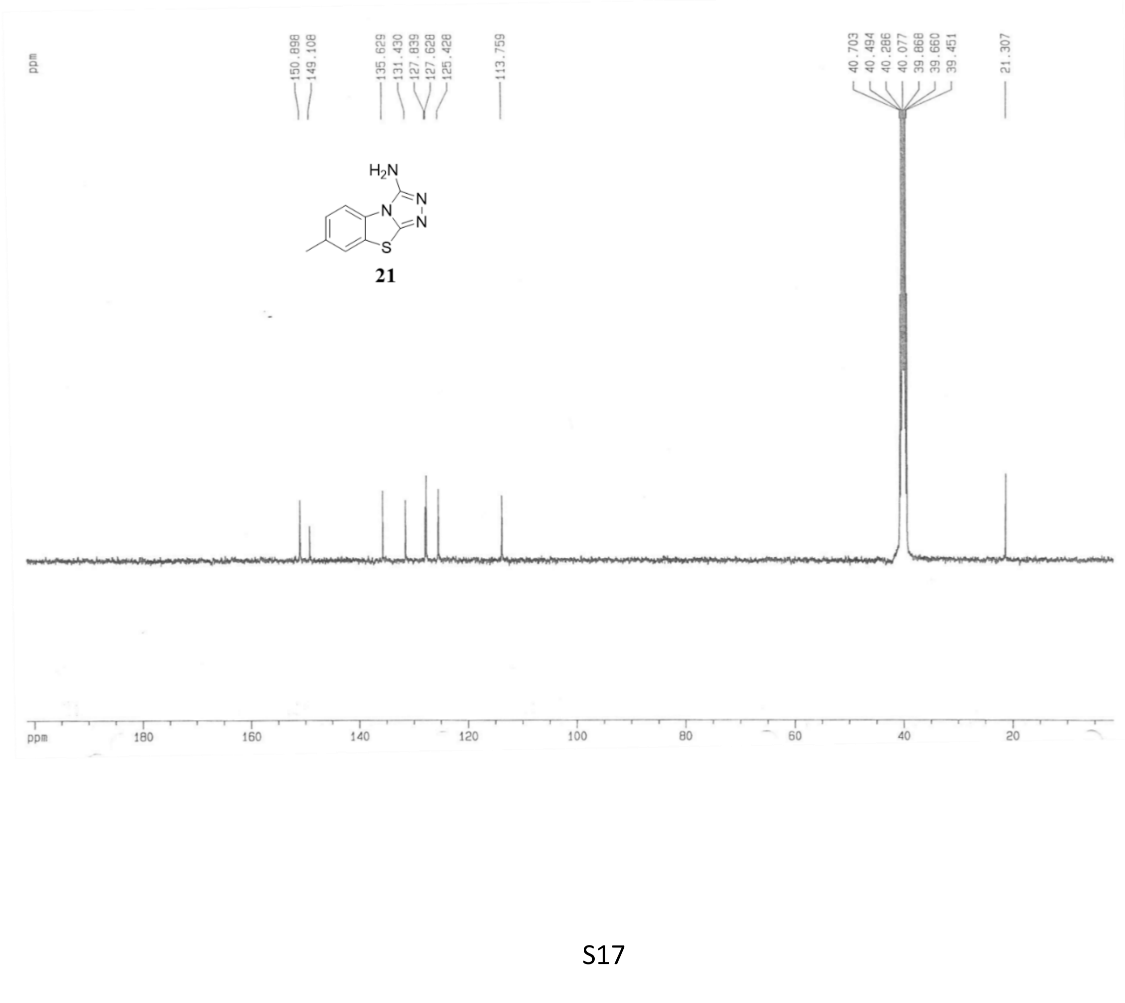
ppm
150.898
149.108
135.629
131.430
127.839
127.628
125.428
113.759
40.703
40.494
40.286
40.077
39.868
39.660
39.451
21.307
H_2N
N
N
N
S
21
ppm
180
160
140
120
100
80
60
40
20

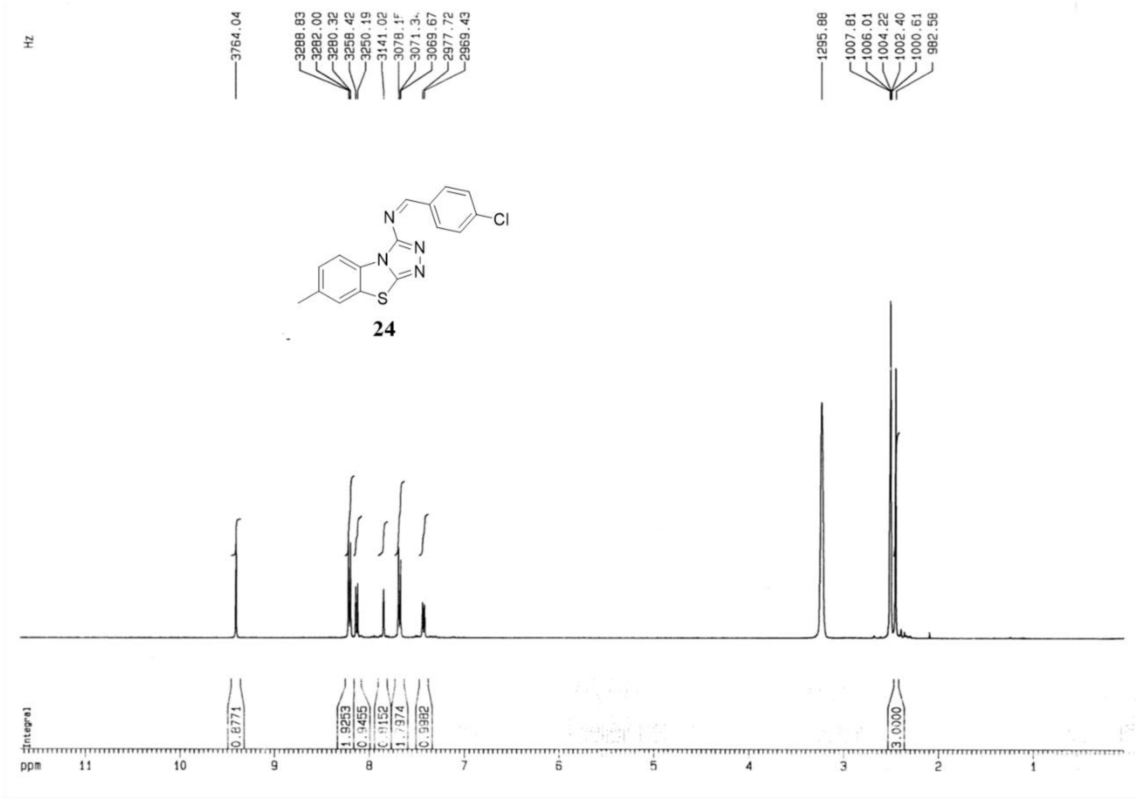
Hz
3764.04
3288.83
3282.00
3280.32
3258.42
3250.19
3141.02
3069.67
2977.72
2969.43
1295.88
1007.81
1006.01
1004.22
1002.40
1000.61
982.58
N
N
N
N
Cl
S
24
Integral
0.8771
1.9253
0.9455
1.7974
0.9982
3.0000
ppm
11
10
9
8
7
6
5
4
3
2
1

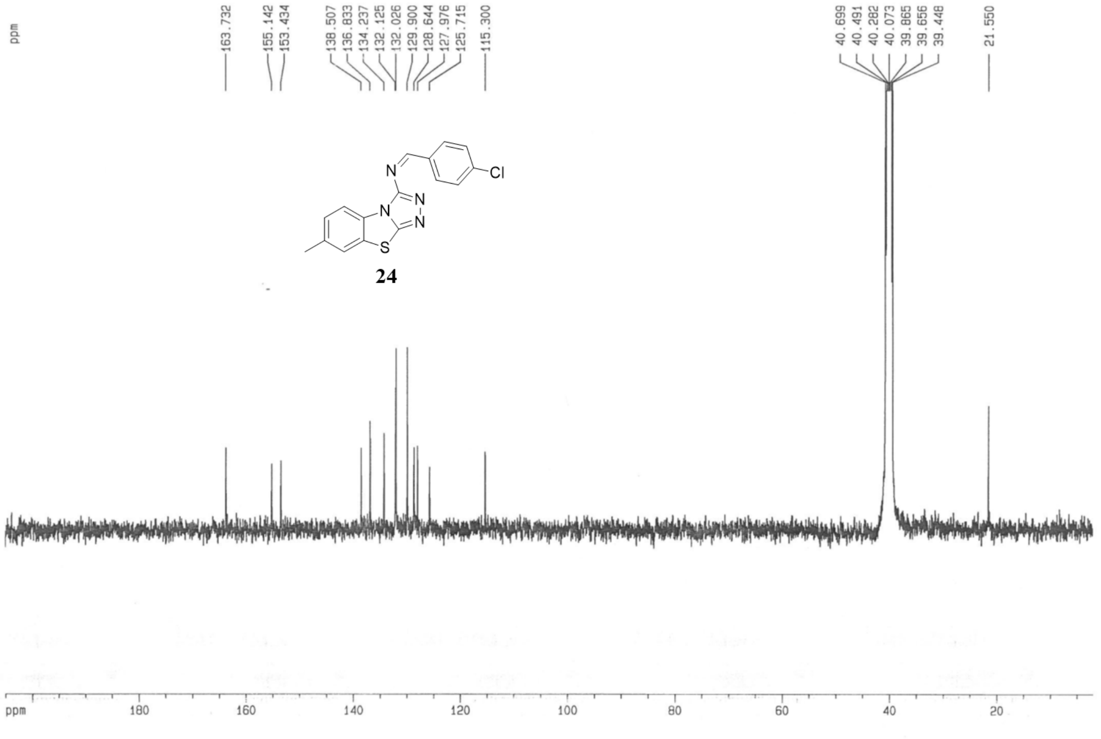
ppm
163.732
155.142
153.434
138.507
136.833
134.237
132.125
132.026
129.900
128.644
127.976
125.715
115.300
40.699
40.491
40.282
40.073
39.865
39.656
39.448
21.550
N
N
N
N
Cl
S
24
ppm
180
160
140
120
100
80
60
40
20

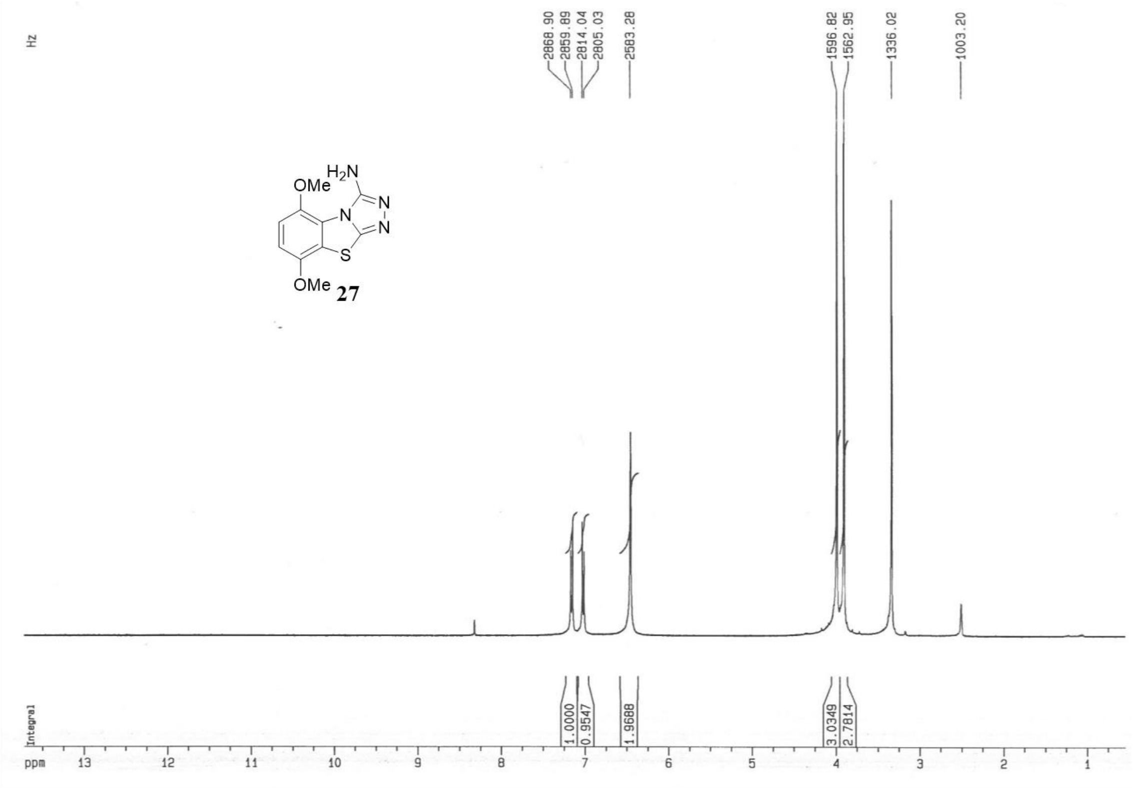
Hz
2868.90
2859.89
2814.04
2805.03
2583.28
1596.82
1562.95
1336.02
1003.20
OMe
H2N
N
N
N
S
OMe
27
Integral
1.0000
0.9547
1.9688
3.0349
2.7814
ppm 13 12 11 10 9 8 7 6 5 4 3 2 1

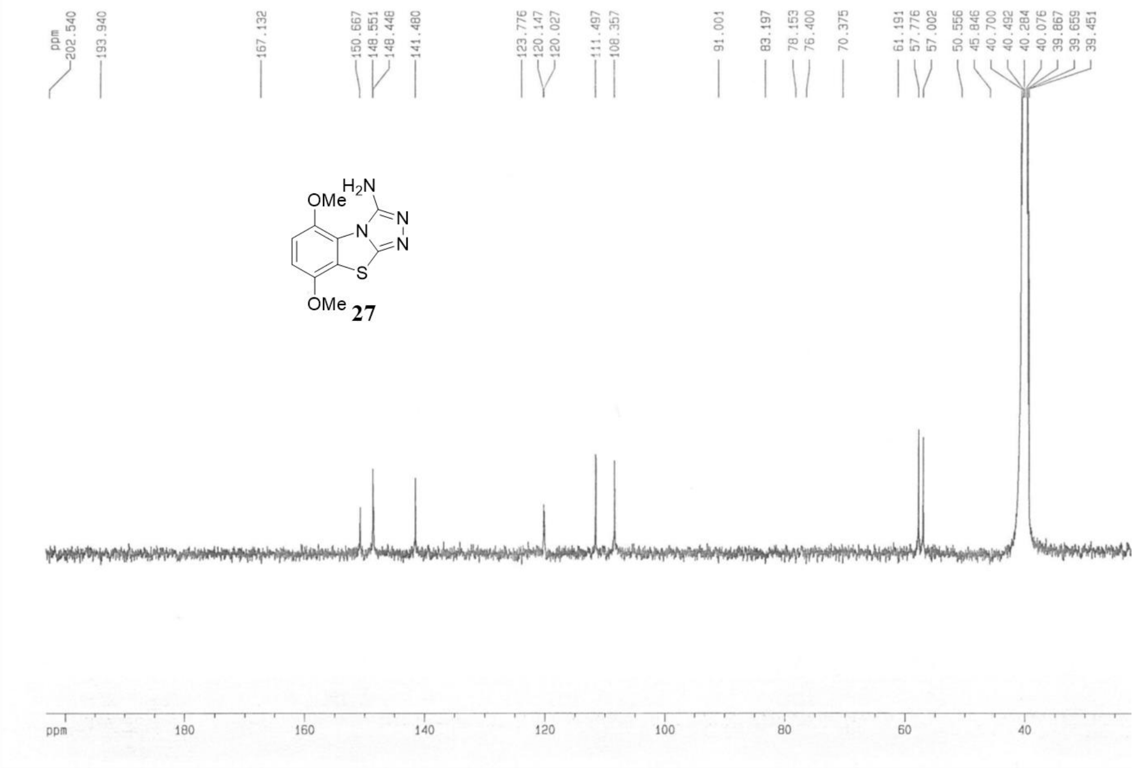
ppm
202.540
193.940
167.132
150.667
148.551
148.448
141.480
123.776
120.147
120.027
111.497
108.357
91.001
83.197
78.153
76.400
70.375
61.191
57.776
57.002
50.556
45.846
40.700
40.492
40.284
40.076
39.867
39.659
39.451
OMe
H2N
N
N
N
S
OMe
27
ppm 180 160 140 120 100 80 60 40

References

(1) Weininger, D. SMILES, a Chemical Language and Information System. 1. Introduction to Methodology and Encoding Rules. *J. Chem. Inf. Comput. Sci.* **1988**, *28* (1), 31–36. https://doi.org/10.1021/ci00057a005.
